# Right Heart Evaluation: A Tough Challenge for Clinicians

**DOI:** 10.3390/life15081194

**Published:** 2025-07-27

**Authors:** Martina Pucci, Luca Maria Capece, Mariateresa Pontoriero, Daniele Paoletta, Marina Iacono, Francesca La Rocca, Roberto Luise, Roberta Esposito

**Affiliations:** 1 Department of Clinical Medicine and Surgery, Federico II University Hospital, 80131 Naples, Italy; martina.pucci@unina.it (M.P.); mariateresa.pontoriero@unina.it (M.P.); d.paoletta@studenti.unina.it (D.P.); marin.iacono@unina.it (M.I.); rob.luise@studenti.unina.it (R.L.); roberta.esposito1@unina.it (R.E.); 2 Department of Clinical and Experimental Medicine, University of Catania, 95122 Catania, Italy; francescalarocca94@hotmail.it

**Keywords:** right ventricle, echocardiography, dyspnea

## Abstract

The right heart–pulmonary circulation unit (RH-PCU) constitutes an integrated anatomo-functional system characterized by high-volume blood flow, low intravascular pressure, and minimal pulmonary vascular resistance. The RH-PCU dysfunction is a challenge for clinicians, as it can result from numerous pathological conditions, each with different clinical presentations. The pathophysiological changes underlying the hemodynamic alterations in the pressure and volume affecting the right ventricle can lead the patient to present with the primary symptom: dyspnea. We review the clinical presentation, the laboratory test, and the role of multimodality imaging in the evaluation of the disfunction of the RHPCU, including echocardiography, stress echocardiography, computed tomography, magnetic resonance imaging, nuclear imaging, and invasive pressure measurement through catheterization. We therefore aimed to describe the various diagnostic options available to clinicians, evaluating their effectiveness and limitations of use.

## 1. Introduction

### 1.1. Pathophysiology

The right heart–pulmonary circulation unit (RH-PCU) constitutes an integrated anatomo-functional system characterized by high-volume blood flow, low intravascular pressure, and minimal pulmonary vascular resistance. Right ventricular (RV) failure is a complex clinical syndrome arising from structural or functional cardiovascular abnormalities that impair the RV’s capacity to adequately fill or effectively eject blood [1]. Etiologies of RV dysfunction can be categorized according to the underlying pathophysiological alteration. The causes of RV dysfunction can be classified based on the type of alteration: volume overload, pressure overload, and a combination of both.

Primary causes of pressure overload include pulmonary hypertension, chronic obstructive pulmonary disease, and adult congenital heart disease. These conditions lead to progressive RV dilation, which impairs the systolic function and reduces the cardiac output, ultimately resulting in RV failure and increased mortality. The most prevalent cause of RV dysfunction, due to an increased afterload, is left-side heart failure (HF), followed by valvular heart disease (VHD) and hypertrophic cardiomyopathy (HCM). The right ventricle (RV) is capable of compensating for the elevated afterload through myocardial hypertrophy and the enhancement of the systolic function.

A mixed pattern of volume and pressure overload is observed in chronic thromboembolic pulmonary hypertension, amyloidosis, and other types of arterial pulmonary obstruction disease.

**Figure 1:**
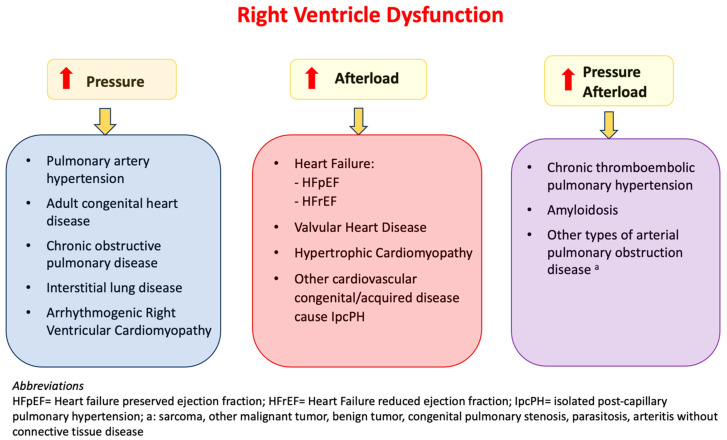
Right ventricular dysfunction physiopathology.

The time of onset, severity, and duration of the damage affect the RV remodeling and failure (Figure 1).

### 1.2. Clinical Presentation

**Figure 2:**
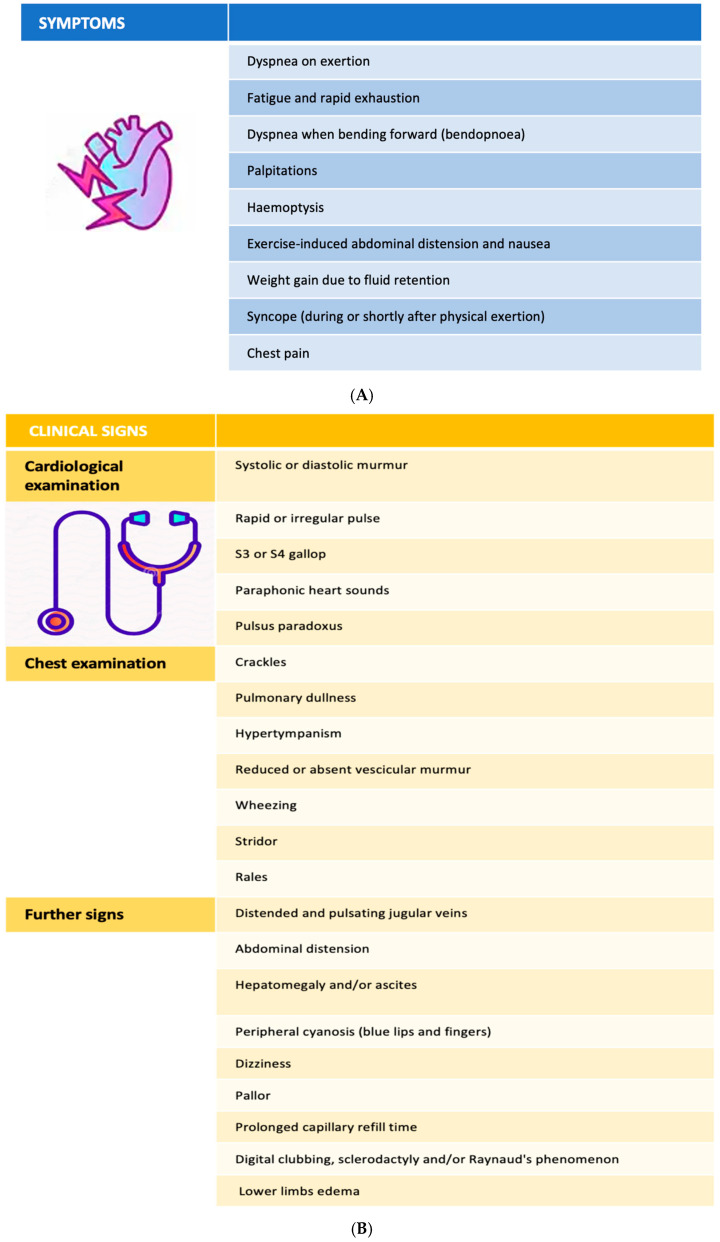

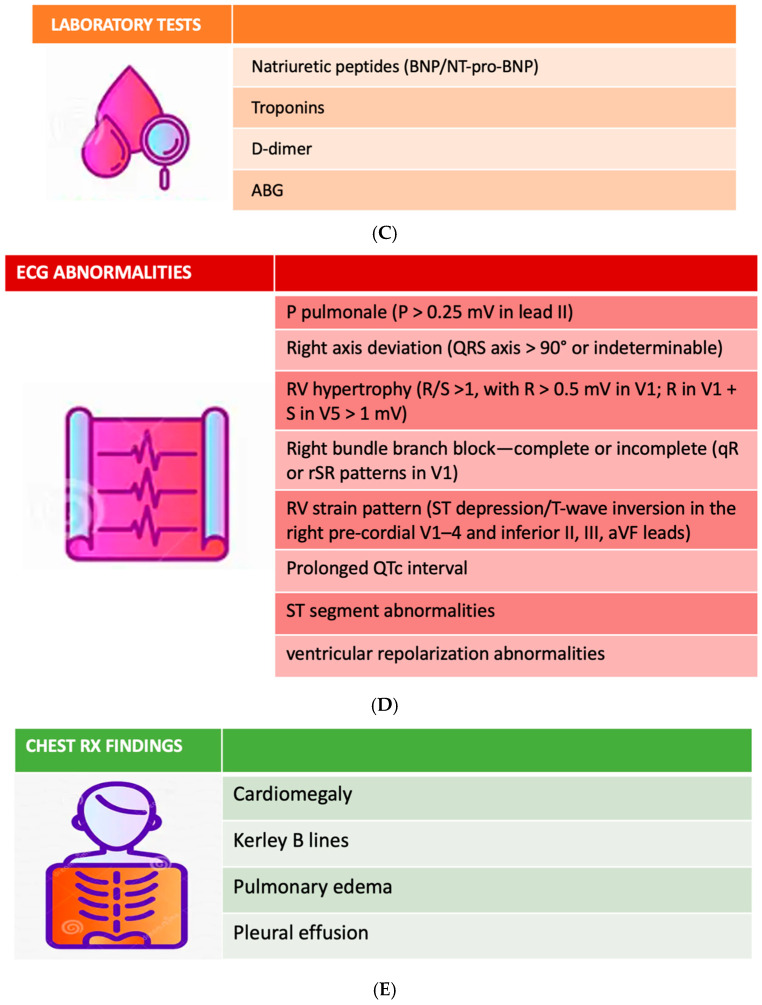
(**A**) Alterations of RH-PCU clinical symptoms. (**B**) Alterations of RH-PCU clinical signs. (**C**) RH-PCU diagnostic algorithm laboratory tests. (**D**) RH-PCU ECG abnormalities. (**E**) RH-PCU Chest RX Findings.

Clinical manifestations of RH-PCU alterations often include dyspnea, accompanied by a range of other signs and symptoms. The diagnostic approach begins with a thorough medical history and physical examination, as specific clinical features can guide suspicion. Patients typically fall into two categories: (1) those with newly onset respiratory symptoms of an undetermined origin and (2) those with pre-existing cardiopulmonary or neuromuscular conditions experiencing symptom worsening. In the former, the goal is to identify a new or occult pathology; in the latter, the goal is to assess the disease progression or the emergence of additional conditions. In all cases, a clinical evaluation remains crucial for the diagnostic orientation (Figure 2).

## 2. Symptoms

The symptoms most frequently accompanying dyspnea include asthenia, bendopnea (dyspnea on forward flexion), palpitations, hemoptysis, fluid retention with consequent weight gain, nausea, exercise-induced abdominal distension, exertional syncope, exertional chest pain, hoarseness, wheezing, and coughing (Figure 2A).

## 3. Signs

Key cardiological findings include the accentuation of the pulmonary component of S2, a systolic murmur from tricuspid regurgitation, a diastolic murmur of pulmonary insufficiency, arrhythmias, an S3 gallop suggestive of LV dysfunction in HF, an S4 gallop associated with hypertensive or ischemic LV dysfunction, paraphonic heart sounds indicative of cardiac tamponade, pulsus paradoxus seen in COPD, asthma, cardiac tamponade, or pericardial constriction.

Chest examinations may reveal crackles consistent with subcutaneous emphysema, dullness to percussion indicating consolidation or pleural effusion, hypertympanism suggestive of pneumothorax or bullous emphysema, a reduced or absent vesicular murmur in pleural effusion or pneumothorax, wheezing indicative of obstructive airway disease, stridor indicative of upper airway obstruction, rales associated with pulmonary edema, pneumonia, or restrictive lung disease.

Additional diagnostic signs include an elevated jugular venous pressure, abdominal distension, hepatomegaly, ascites, peripheral cyanosis, dizziness, pallor, cold extremities, a prolonged capillary refill time, digital clubbing, sclerodactyly, Raynaud’s phenomenon, lower limb edema, and clinical signs of deep vein thrombosis (Figure 2B).

### Diagnosis

The RH-PCU diagnostic algorithm includes laboratory tests, electrocardiograms, chest X-rays, lung ultrasounds, chest computed tomography, standard echocardiography, stress echocardiography, nuclear imaging tests, and right heart catheterization.

## 4. Lab Test

The biomarkers to be investigated are BNP/NT-proBNP, troponins, D-dimer, and the ABG (arterial blood gas) analysis. However, these biomarkers, although very useful in the diagnostic orientation, are often not very specific (Figure 2C). [NT-ProBNP: a cobas e411 analyser (Roche Diagnostics, Basel, Switzerland); Troponins: electrochemiluminescence immunoassay on Elecsys 2010 analyzer (Roche Diagnostics); D-Dimer: enzyme-linked immunosorbent assays (ELISA), immunofluorescent assays, and latex agglutination assays].

## 5. ECG

The electrocardiographic anomalies to look for are as follows: P pulmonale (amplitude greater than 0.25 mV in lead II), right axis deviation (QRS axis > 90° or indeterminable), a prolonged QTc interval, a complete or incomplete right bundle branch block (qR or rSR patterns in V1), an RV strain pattern (ST depression/T-wave inversion in the right pre-cordial V1–4 and inferior II, III, aVF leads), RV hypertrophy (R/S > 1, with R > 0.5 mV in V1; R in V1 + S in lead V5 > 1 mV), ST segment abnormalities, and ventricular repolarization abnormalities (Figure 2D).

## 6. Chest X-Ray

The radiographic signs to look for are right heart enlargement, pulmonary artery enlargement, left atrial enlargement, left ventricular dilatation, the presence of Kerley B lines, pleural effusion, pulmonary edema, pneumothorax, the flattening of the diaphragm, atelectasis, and signs of a phlogistic pathology (Figure 2E).

## 7. Lung Ultrasound

In the evaluation of the RH-PCU, a lung ultrasound (LUS) can complement echocardiography by providing additional insights into various pathological conditions affecting the pulmonary parenchyma [2].

Key ultrasonographic findings include the following:

- Lung sliding: Dynamic shimmering at the pleural line, producing the characteristic *seashore sign* on M-mode imaging.
- Lung point: The transition zone where normal lung sliding intermittently appears, typically during inspiration, which is pathognomonic for a pneumothorax.
- A-lines: Horizontal reverberation artifacts parallel to the pleural line, suggestive of a normally aerated lung.
- B-lines: Vertical, hyperechoic artifacts originating from the pleural line associated with interstitial syndrome.
- Pleural effusion: An anechoic or hypoechoic fluid collection in the pleural space [3].

In patients with pulmonary hypertension due to left heart disease, B-lines, the sonographic sign of pulmonary interstitial syndrome, can be detected [4]. B-lines reflect the extent of extravascular lung water (cardiogenic B-lines) and may serve as a valuable tool for the diagnosis, monitoring, and prognostic assessment of patients with heart failure (HF) [5]. Traditionally assessed using chest radiography or advanced imaging techniques, this parameter can now be evaluated non-invasively and in real-time with a LUS.

In contrast, in patients with pulmonary hypertension secondary to interstitial lung disease, B-lines may reflect the degree of pulmonary interstitial fibrosis (referred to as pneumogenic B-lines). Pneumogenic B-lines can be distinguished from cardiogenic B-lines primarily by alterations in the appearance of the pleural line: in heart failure, the pleural line is generally smooth and thin, whereas in pulmonary fibrosis it tends to be thickened and irregular [6].

The LUS also enables the detection of other pulmonary pathological conditions, such as the presence of pneumonia, pleural effusion, lung consolidations, acute respiratory distress syndrome (ARDS), and even pneumothorax (PNX).

In ARDS and pneumonia, the inflammatory exudate acts as an adhesive, anchoring the lung to the parietal pleura and thereby eliminating lung sliding. The pleural line often appears thickened and irregular, potentially due to the presence of a subpleural alveolar syndrome. These static sonographic findings are typically accompanied by a key dynamic abnormality: the absence of or marked reduction in lung sliding. This feature is particularly useful in distinguishing B-lines associated with ARDS or pneumonia from those seen in cardiogenic acute pulmonary edema.

Lung consolidation represents the advanced stage of interstitial syndrome, characterized by the transition to a consolidated lung appearance. On ultrasound, the lung becomes echogenic with a tissue-like pattern resembling that of the liver or spleen. This sonographic appearance reflects the replacement of alveolar air with non-aerated material, typically fluid, pus, or blood.

In a PNX condition we first detect abolished lung sliding. The detection of lung sliding effectively rules out a pneumothorax at the site of the ultrasound examination. Another pathognomonic sign to be detected is the lung point: a point where the probe is positioned over the presumed interface between the pneumothorax and the aerated lung, and the abrupt reappearance of lung signs, such as lung sliding and B-lines (lung rockets), typically occurs during inspiration [7].

A pulmonary ultrasound is certainly very useful considering its advantages, such as its bedside applicability, rapid execution, and low costs. However, computed tomography remains the gold standard for the diagnosis of lung diseases. 

## 8. Computed Tomography

Chest computed tomography is a critical imaging modality in the evaluation of the RH-PCU [8].

In cases of suspected pulmonary hypertension (PH), characteristic CT findings include the dilatation of the main pulmonary artery, right atrial enlargement, right ventricular (RV) hypertrophy and dilatation, the abrupt tapering of peripheral pulmonary arteries, bronchial artery enlargement, and a mosaic attenuation pattern reflective of heterogeneous lung perfusion [9].

CT pulmonary angiography (CTPA) offers a superior sensitivity compared to conventional invasive angiography for detecting both central and peripheral pulmonary artery emboli [10].

High-resolution computed tomography (HRCT) plays a crucial role in the diagnosis of interstitial lung disease (ILD) and chronic obstructive pulmonary disease (COPD), with or without coexisting pulmonary hypertension (PH). In ILD, characteristic HRCT findings include peripheral interlobular septal thickening, ground-glass opacities, traction bronchiectasis, and honeycombing [11].

## 9. Standard Echocardiography

Transthoracic Doppler echocardiography (TDE) is a commonly employed modality for the assessment of the RH-PCU, offering the advantages of wide availability, cost-effectiveness, and excellent patient tolerability. It enables the rapid estimation of pulmonary hemodynamics and provides essential etiological and prognostic insights across a range of clinical settings.

The first step is to evaluate all possible diseases affecting the left heart that can cause, due to an increase in the afterload, the dysfunction of the RH-PCU.

- ***Left Heart Disease Assessment***

Hypertension induces LV hypertrophy and eventual failure, which subsequently leads to the upstream transmission of an elevated LV end-diastolic pressure, increased pulmonary vascular pressures, pulmonary vasoconstriction, and vascular remodeling, ultimately resulting in RV failure in a subset of patients. RV function is impaired in the setting of hypertension, primarily as a consequence of LV remodeling and elevated pulmonary vascular resistance (PVR). These alterations lead to abnormal diastolic function parameters and, in a subset of patients, reduced systolic performance indices [12]. RV dysfunction can be observed in the context of both left-sided and right-sided valvular heart disease [13]. The diagnosis of pulmonary hypertension (PH) related to valvular heart disease (VHD) is based on the following criteria: a mean pulmonary arterial pressure (PAP) greater than 25 mmHg associated with pulmonary capillary wedge pressure (PCWP) or a left ventricular (LV) end-diastolic pressure greater than 15 mmHg. In VHD, an increased left ventricular (LV) volume or pressure leads to an elevated left atrial (LA) pressure, resulting in passive backward transmission to the pulmonary venous system, which can trigger vascular vasoconstriction and hyperplasia, thereby contributing to the progression of PH, resulting in RV failure [14]. Therefore, the evaluation of the RH-PCU is crucial for the detection of left heart valvular diseases.

Another type of disease to be investigated is cardiomyopathies (CM), a heterogeneous group of cardiac disorders characterized by a primitive disorder of the heart muscle which are not secondary to valvulopathies, respiratory disease, or coronary artery disease (CAD), divided into three main categories: dilated cardiomyopathy (DCM), hypertrophic cardiomyopathy (HCM), and restrictive cardiomyopathy (RCM). The primary mechanisms underlying RV dysfunction in DCM include a reduced LVEF, elevated LV filling pressures, and the retrograde transmission of these elevated pressures to the pulmonary circulation. Additionally, RV function may be impaired by the direct extension of the myopathic process to RV cardiomyocytes, interventricular septal dysfunction—which compromises RV contractility due to ventricular interdependence—and reduced RV diastolic filling, which results from both LV dilation and limited RV distensibility [15].

RH involvement is also frequently observed in HCM, where it plays a key role in the pathophysiology of major clinical manifestations such as HF and arrhythmias. In HCM, the RV involvement is recognized as a critical contributor to the development and clinical manifestation of heart failure alongside supraventricular arrhythmias, and it may also be implicated in the pathogenesis of sudden cardiac death (SCD). Right ventricular hypertrophy (RVH) occurs in about 50% of patients and may result from the primary myocardial disease itself, altered afterload conditions (secondary to LV dysfunction and PH), or ventricular interdependence resulting from the hypertrophy of the interventricular septum, which is shared by both ventricles. RV systolic and diastolic dysfunctions are also commonly observed in HCM.

The RCM group encompasses a range of conditions, including infiltrative diseases (e.g., amyloidosis, sarcoidosis), characterized by the primary involvement of the interstitial space; storage disorders (e.g., Fabry disease, hemochromatosis, Gaucher disease, and glycogen storage diseases), defined by the intracellular accumulation of metabolic products; and endomyocardial diseases, in which restriction is primarily due to fibrotic changes in the endocardium, as seen in carcinoid heart disease and endomyocardial fibrosis. Also, in Restrictive Cardiomyopathies we can observe RV dysfunction: for example, RVH is frequent in Fabry disease; furthermore, it has been demonstrated that with advanced echocardiographic techniques in these patients, there is a subclinical dysfunction of the right ventricle even in the early stages of the disease [16].

Acute RV failure, characterized as a rapidly progressive syndrome marked by systemic congestion due to impaired RV filling and/or a decreased RV output, can ultimately be observed in right ventricular myocardial infarction (RVMI). HF following RVMI is classically observed after the acute occlusion of the proximal right coronary artery, but RV dysfunction may also occur after larger infarctions in the left anterior descending (LAD) artery. Therefore, when evaluating the RH-PCU using standard echocardiography, particular attention must be paid to regional wall motion abnormalities, which can indicate segmental RV involvement [17].

- ***Right Heart Size and Function Assessment***

According to recent clinical guidelines [18], a holistic approach is recommended for evaluating the RH-CPU. This should include both standard and advanced echocardiographic techniques, allowing for a multidimensional assessment of the right heart structure and function. The RV is located anteriorly, immediately behind the sternum in the normal heart [19], which makes the assessment challenging [20].

The assessment of the RH-PCU should consider the morphology of the RV from multiple views (parasternal, apical, and subcostal) to estimate the thickness of the RV wall and the RA and RV area, integrating, when is feasible, the 3D method to obtain the volume data.

RV dysfunction or an increased overload may result in an increase in the size of the right chambers.

Furthermore, the assessment should incorporate an evaluation of the inferior vena cava (IVC) for size and respiratory variations as well as the presence of pericardial effusion, both of which offer indirect insights into right-sided filling pressures.

The functional assessment of the RV can be achieved through a variety of quantitative parameters, including the following: tricuspid annular systolic plane excursion (TAPSE), RV fractional area change (FAC), the systolic velocity (S′) of the lateral tricuspid annulus by tissue Doppler imaging (TDI), longitudinal speckle-tracking echocardiographic strain, Right Ventricular Index of Myocardial Performance (RIMP), and RV myocardial work.

Among these, the TAPSE is one of the most widely used due to its simplicity and reproducibility. It reflects the longitudinal displacement of the lateral tricuspid annulus during systole, measured by M-mode echocardiography. However, its diagnostic accuracy can be limited by its load dependence and angle dependency and therefore should be interpreted within the broader clinical and imaging context. RV dysfunction can be associated with a reduced TAPSE (<17 mm) [21]. It has also been demonstrated that the TAPSE serves as a robust prognostic marker in cardiovascular diseases, especially in PH [22,23].

The RV systolic wave velocity (S′), a widely used and practical method for assessing myocardial velocities at the tricuspid annulus, serves as an indicator of the global RV systolic function; an S′ value < 9.5 cm/s is considered abnormal. S′ limits are angle- and load-dependent. Therefore, for an accurate assessment of RV function, it should not be used in isolation because it can underestimate the RV function.

The RIMP, also known as the Tei index, is an index of the global RV performance and represents the ratio between the isovolumetric contraction and relaxation times relative to the ejection time. This index provides insight into both the systolic and diastolic function, although it is also influenced by loading conditions and the heart rate.

The FAC is the result of the difference between the end-diastolic area and the less end-systolic area, divided by the end-diastolic area; a value less than 35% is considered abnormal [21].

Three-dimensional echocardiography (3DE) enables the accurate and reproducible assessment of the RV volumes and EF [21]. The quantification of the right ventricular (RV) size and function using three-dimensional echocardiography (3DE) is not limited by the geometric assumptions or imaging plane misalignment, offering a superior accuracy over conventional echocardiography indices when the image quality is adequate [24]. The 3DE-derived RVEF closely correlates with MRI [25].

Strain is a dimensionless measurement, describing the deformation of the myocardium that occurs during the cardiac cycle. Strain imaging, whether assessed globally or segmentally, provides an objective quantification of the myocardial deformation and offers valuable insights into the myocardial functional status. It is expressed as a negative percentage [26]. The longitudinal free wall RV strain quantifies the longitudinal motion of the RV free wall, which represents the predominant component of the RV systolic ejection. Strain is more sensitive than the EF in detecting early or subclinical myocardial dysfunction, making it a valuable tool for long-term monitoring in various patient populations [27].

Strain is limited by suboptimal acoustic windows, and, given that speckle-tracking echocardiography relies on a single cardiac cycle strain analysis, it is not feasible to perform accurate myocardial deformation assessments in patients with arrhythmias [28].

Right ventricular myocardial work (RVMW) is a non-invasive technique for evaluating RV function through the analysis of RV pressure–strain loops. This approach offers a comprehensive assessment of the RV systolic performance and demonstrates a stronger correlation with the invasively measured stroke volume and stroke volume index compared to conventional echocardiographic parameters [29]. RVMW integrates the RV GLS, pulmonary artery pressure, and cardiac cycle timing events, thereby offering a more accurate evaluation of RV systolic function than conventional measures. It is considered a reliable and reproducible marker for assessing the RV systolic performance [30].

- ***Hemodynamics Parameter Assessment***

RV diastolic function includes the tricuspid E/A ratio, the E/e’ ratio, the E-wave deceleration time (normal range 120–229 ms), and the assessment of the hepatic vein flow. The E/A ratio indicates the relationship between the relaxation of the right ventricle (E wave) and the atrial contraction (A wave) that occurs in the late phase of the diastole. An E/A ratio (<0.8) with an increased deceleration time represents impaired ventricular relaxation; instead, an E/A ratio > 2.1 with a decreased deceleration time (<120 ms) represents restrictive physiology—a late phase of diastolic dysfunction [31].

The E-wave deceleration time reflects the rate of the pressure equalization between the atrium and ventricle following the onset of early diastolic filling. In adults, shorter deceleration times (typically 160–240 ms) are indicative of a more compliant ventricle [32]. Hemodynamic parameters can be estimated non-invasively using Doppler echocardiography. The peak tricuspid regurgitation velocity (TRV) is defined as the highest modal systolic velocity measured at the leading edge of the continuous-wave Doppler spectral envelope. In the absence of flow obstruction between the right ventricle and pulmonary artery, the TRV will have a linear positive correlation with the systolic pulmonary artery pressure (sPAP). Flow velocities are directly proportional to the flow volume and inversely proportional to the cross-sectional area of the regurgitant orifice. Consequently, in cases of right ventricular or tricuspid annulus dilation (resulting in an increased regurgitant orifice area) and/or an impaired RV function or contractility, the positive correlation between the TRV and sPAP is attenuated [33].

A TRV greater than 2.8 m/s may indicate the presence of PH; however, the TRV alone is insufficient for a definitive diagnosis. Additional echocardiographic parameters assessing the RV morphology and function are necessary to accurately estimate the probability of PH [34].

The TAPSE/PASP ratio serves as a valuable clinical index reflecting the length-to-force relationship, enabling a more precise assessment of the RV function in patients with heart failure and an enhancing prognostic stratification (high: >0.32 mm/mmHg; middle: 0.19–0.32 mm/mmHg; low: <0.19 mm/mmHg).

**Figure 3:**
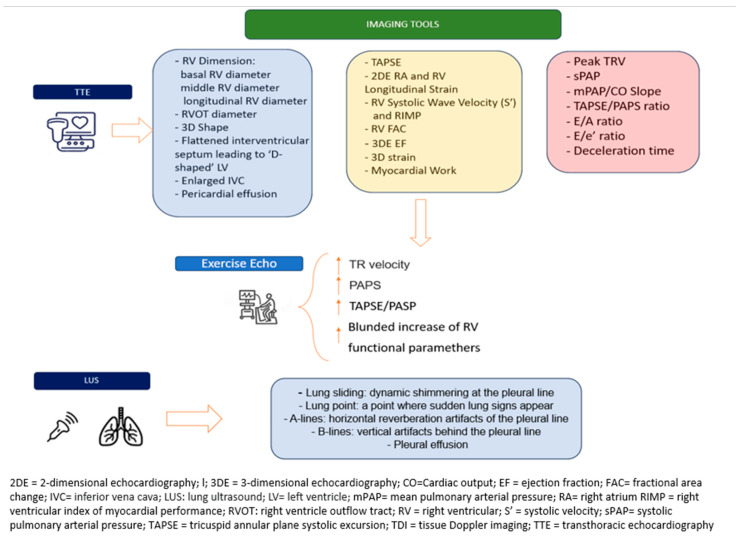
RH-PCU imaging tools.

Additionally, the mean pulmonary artery pressure to cardiac output ratio (mPAP/CO slope) is an important hemodynamic parameter, particularly in the context of exercise testing. A slope >3 mmHg·min/L is associated with exercise-induced PH and represents both diagnostic and prognostic significance [35] (Figure 3).

## 10. Stress Echocardiography

Stress echocardiography, a well-established technique for the diagnosis and prognostic stratification of coronary artery and valvular diseases, has seen increasing application over recent decades in the evaluation of the RH-PCU.

The physiological response to moderate exercise involves a mild increase in the mean pulmonary artery pressure (mPAP) that correlates linearly with the cardiac output (CO), accompanied by a decrease in the pulmonary vascular resistance (PVR) due to the dilation of compliant small vessels and/or the recruitment of additional vessels, primarily in the upper regions of the normal lung [36].

Normal values of Doppler-derived sPAP in normal subjects during exercise rarely exceed 40 mmHg, whereas in athletes, values reach 60 mmHg [37].

Notably, the PVR exhibits considerable interindividual variability. As a result, the rise in the mean pulmonary arterial pressure (mPAP) relative to the CO during exercise is highly variable, with reported mPAP/CO slopes ranging from 0.5 to 3.0 mmHg per L/min [38].

Patients exhibiting an elevated mPAP beyond normal ranges warrant a comprehensive clinical evaluation to identify underlying causes of exercise-induced PH, including left heart disease, pulmonary parenchymal disorders, PAH, and CTEPH. However, while in the settings of coronary artery disease, valvular heart disease, and diastolic function, stress echocardiography underwent a safety and prognostic validation [39], and the non-invasive evaluation of the right side of the heart by stress echocardiography is not yet standardized and there is a very important knowledge gap in studying the impact of exercise on the right-sided circulation.

**Figure 4:**
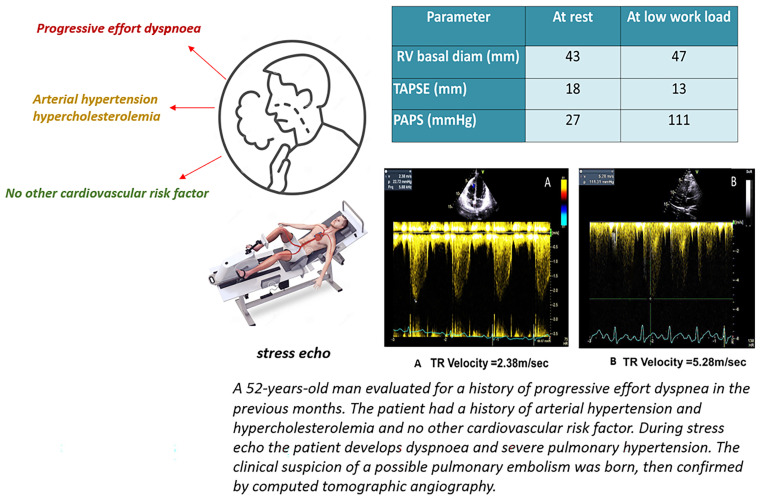
Clinical case description of patients with dyspnea. (**A**) TR Velocity at rest; (**B**) TR Velocity during stress echo.

A recent multicenter cohort study involving 2228 participants, conducted by the RIGHT Heart International NETwork (RIGHT NET), investigated the physiological versus pathological responses of the right ventricle and pulmonary circulation to exercise. The study population included 375 healthy controls, 40 athletes, 516 individuals with cardiovascular risk factors, 17 patients with pulmonary arterial hypertension (PAH), 872 patients with connective tissue diseases without overt pulmonary hypertension, 113 with left-sided heart disease, 30 with lung disease, and 265 individuals chronically exposed to high altitudes. Gargani et al. were the first to non-invasively define the normal reference ranges for the mean pulmonary arterial pressure to cardiac output (mPAP/CO) ratio, reporting values between 1.8 and 5.5 mmHg/L/min at rest and between 0.9 and 4.0 mmHg/L/min during peak exercise. They also identified the normal range for the mPAP/CO slope as 0.2 to 3.5 mmHg/L/min and proposed a prognostic cutoff value above 5 mmHg/L/min, which was associated with increased all-cause mortality across various disease categories. Additionally, Gargani et al. established normal values for the tricuspid annular plane systolic excursion to systolic pulmonary artery pressure (TAPSE/sPAP) ratio, ranging from 0.7 to 2.0 mm/mmHg at rest and from 0.5 to 1.5 mm/mmHg at peak exercise. However, prognostic significance was demonstrated only for resting values, with a TAPSE/sPAP ratio < 0.7 mm/mmHg being associated with worse outcomes. Taken together, these findings enhance our understanding of the clinical relevance and physiological complexity of right ventricular function and its coupling with the pulmonary circulation. Given their simplicity and quantitative nature, these parameters should become part of every echocardiographic study, and they have the potential to be new tools to help with the diagnosis of latent or overt pulmonary hypertension with or without an altered RV function adaptation to increased loading conditions [40] (Figure 4).

## 11. Cardiac Magnetic Resonance

Cardiac magnetic resonance (CMR) provides a precise assessment of the right ventricular anatomy, function, and tissue characterization [41].

CMR is considered the gold standard for evaluating the RV size and function. CMR uses steady-state free precession ‘cine’ images [42] to evaluate the RV anatomy and can discriminate anatomical and regional wall motion abnormalities. CMR enables the assessment of the blood flow in the pulmonary artery, aorta, and vena cava, allowing for the accurate quantification of the stroke volume (SV).

The anatomical abnormalities that CMR can identify are the RV dilatation and hypertrophy [43], IVS straightening [44], RA enlargement, and PA dilatation [45]. CMR can estimate the RV volume through Simpson’s method, calculating the total volume by the summation of the endocardial board in multiple slices. Four-dimensional cardiovascular magnetic resonance flow imaging (4D Flow CMR) enables the comprehensive and precise evaluation of blood flow dynamics within a single acquisition. Four-dimensional flow CMR is an extension of 2D flow CMR [46]. It enables the simultaneous quantification and visualization of hemodynamic abnormalities and morphological alterations. In addition to conventional 2D phase-contrast (PC) parameters, such as the flow direction, forward flow volume, reverse flow volume, regurgitant fraction, and peak velocity, 4D flow MRI provides a comprehensive retrospective functional assessment of blood flow dynamics and enables the detailed visualization of complex flow patterns [47] While primarily utilized in the evaluation of congenital heart disease (CHD), 4D flow MRI has also demonstrated significant clinical utility in a variety of other cardiovascular conditions, including aortic aneurysms, aortic stenosis, pulmonary hypertension, and valvular heart disease [48]. CMR with 4D flow imaging enables a comprehensive assessment of the PAH severity and can be utilized to monitor the disease progression and therapeutic response [49].

RV strain imaging is a CMR technique that measures the deformation of a myocardial segments. Feature-tracking technology is a post-processing technique applicable to routinely acquired cine MR images [50]; it operates by identifying distinct features within the images and tracking their displacement across successive frames in the sequence [51].

Artifacts caused by through-plane motion represent a primary limitation, as features that move out of the imaging plane cannot be reliably tracked [52].

The quantification of the RV strain using CMR-FT is feasible in most patients, correlates with the disease severity, and independently predicts poor outcomes in pulmonary hypertension (PH). This is also associated with an impaired RVEF [53].

Late gadolinium enhancement (LGE) is the most valuable CMR technique for myocardial tissue characterization and plays a central role in both the diagnostic evaluation and prognostic stratification of cardiomyopathies [54]. LGE is a marker of myocardial fibrosis. The distribution pattern of LGE enables a highly accurate differentiation between ischemic and non-ischemic heart disease. Moreover, in the context of various cardiomyopathies, specific LGE patterns can aid in establishing the underlying diagnosis. The LGE occurs at the RV insertion point (RVIP) in patients with PH, and it is a marker for more advanced diseases and poor prognoses [46] but was not associated with an increase in overall mortality [55].

T1 mapping quantifies the longitudinal (spin–lattice) relaxation time, whereas contrast-enhanced T1 mapping, used in conjunction with native T1 values, is primarily employed to calculate the ECV fraction. Areas of fibrosis and scar tissue demonstrate reduced T1 relaxation times following the contrast administration.

T_1_ times did not predict mortality in a large cohort of patients affected by PH [56].

The ECV serves as a biomarker of myocardial tissue remodeling. An increased extracellular volume (ECV) was associated with RV dilation, increased stiffness, and impaired RV strain [57].

## 12. Cardiac Computed Tomography and Nuclear Imaging

Cardiac CT provides an accurate quantification of the RV volume and function. Images are acquired with ECG synchronization, using either retrospective gating or prospective triggering, to minimize motion artifacts, following the intravenous administration of iodinated contrast agents. CT may serve as an alternative modality for assessing the cardiac chamber function in patients who are contraindicated for CMR or when it is not available and the image quality of the echocardiography is not high. CT provides accurate and reproducible assessments of the right ventricular (RV) morphology and function [58] but overestimates the volumes [59] and RVEF [60] by varying degrees.

Nuclear imaging techniques, such as hybrid CMR/FDG-PET, provide an assessment of the RV perfusion, metabolism, morphology, and EF. These modalities have emerged as clinically valuable tools for evaluating RV perfusion and metabolism, as well as for detecting isolated RV infarction or left ventricular (LV) infarction with RV involvement [18]. Gated blood pool single-photon emission computed tomography (SPECT) does not rely on geometric assumptions and enables the quantification of both the global and regional RV function, demonstrating a good diagnostic accuracy when compared to CMR [61].

**Figure 5:**
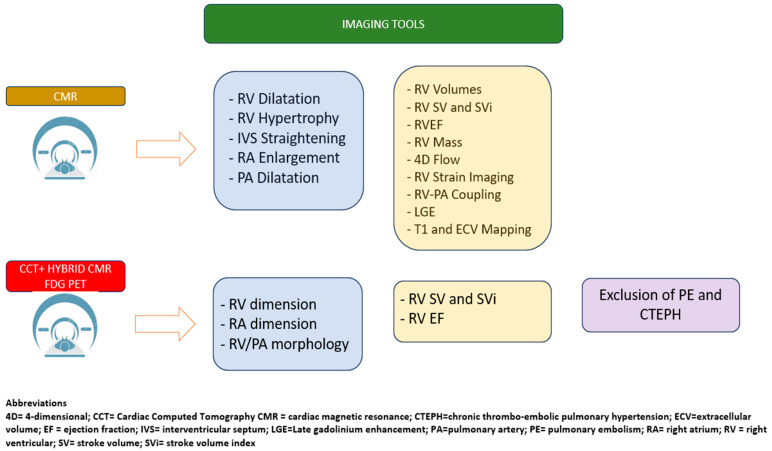
RH-PCU advanced imaging tools.

Nuclear imaging is not considered a first-line modality for the assessment of the RV function, but it may serve as an alternative when other imaging techniques are unavailable or contraindicated (Figure 5).

## 13. Right Heart Catheterization

Right heart catheterization (RHC) is an invasive hemodynamic technique that enables the direct measurement of right-sided cardiac pressures and the calculation of the cardiac output. Pulmonary artery catheterization aids in the diagnosis and management of diverse cardiovascular conditions, including pulmonary hypertension, cardiogenic shock, mixed shock states, cardiac tamponade, and mechanical complications related to ST-segment elevation myocardial infarctions. RHC remains the gold standard for the diagnosis and classification of PH. For a comprehensive evaluation of cardiopulmonary hemodynamics, all these parameters must be measured: the right atrial pressure (RAP), systolic pulmonary artery pressure (sPAP), diastolic pulmonary artery pressure (dPAP), mean pulmonary artery pressure (mPAP), pulmonary arterial wedge pressure (PAWP), cardiac output (CO), mixed venous oxygen saturation (SvO_2_), arterial oxygen saturation (SaO_2_), and systemic blood pressure. In addition to diagnosing and classifying PH, clinical indications include hemodynamic assessments of the heart [62]. RHC performs vasoreactivity tests. Pulmonary vasoreactivity testing is performed to identify patients with idiopathic, heritable, or drug-induced PAH who may benefit from high-dose calcium channel blocker (CCB) therapy [63].

## 14. Conclusions

Historically considered a passive conduit between the systemic venous system and the pulmonary circulation, the right heart is now recognized as having a significant hemodynamic role. The current understanding highlights a close hemodynamic interdependence between the right ventricle, pulmonary circulation, and left ventricle. The RV plays a crucial role in adapting to various physiological and pathological stresses. During dynamic exercise, the “right heart–pulmonary circulation unit” undergoes considerable stress and, in healthy individuals, responds with an increased RV contractility and cardiac output, a decreased PVR, and only a modest rise in the pulmonary artery pressure. Conversely, in patients with cardiac and/or pulmonary diseases, such as ischemic heart disease, HF, severe valvular stenosis or insufficiency, systemic sclerosis, or COPD, the RV contractile reserve may be impaired, leading to an abnormal elevation of the pulmonary systolic blood pressure during exercise. These observations underscore the critical role of multimodality imaging in the assessment of the RH-PCU. The diagnostic algorithm should incorporate lung ultrasounds, computed tomography (CT), standard and advanced echocardiography, nuclear imaging, and invasive techniques such as right heart catheterization.

Notably, exercise echocardiography provides unique diagnostic and prognostic information, enabling the early identification of RV dysfunction or PH, the clarification of the underlying etiology, and informed decision-making on the timing of therapeutic interventions.

**Figure 6:**
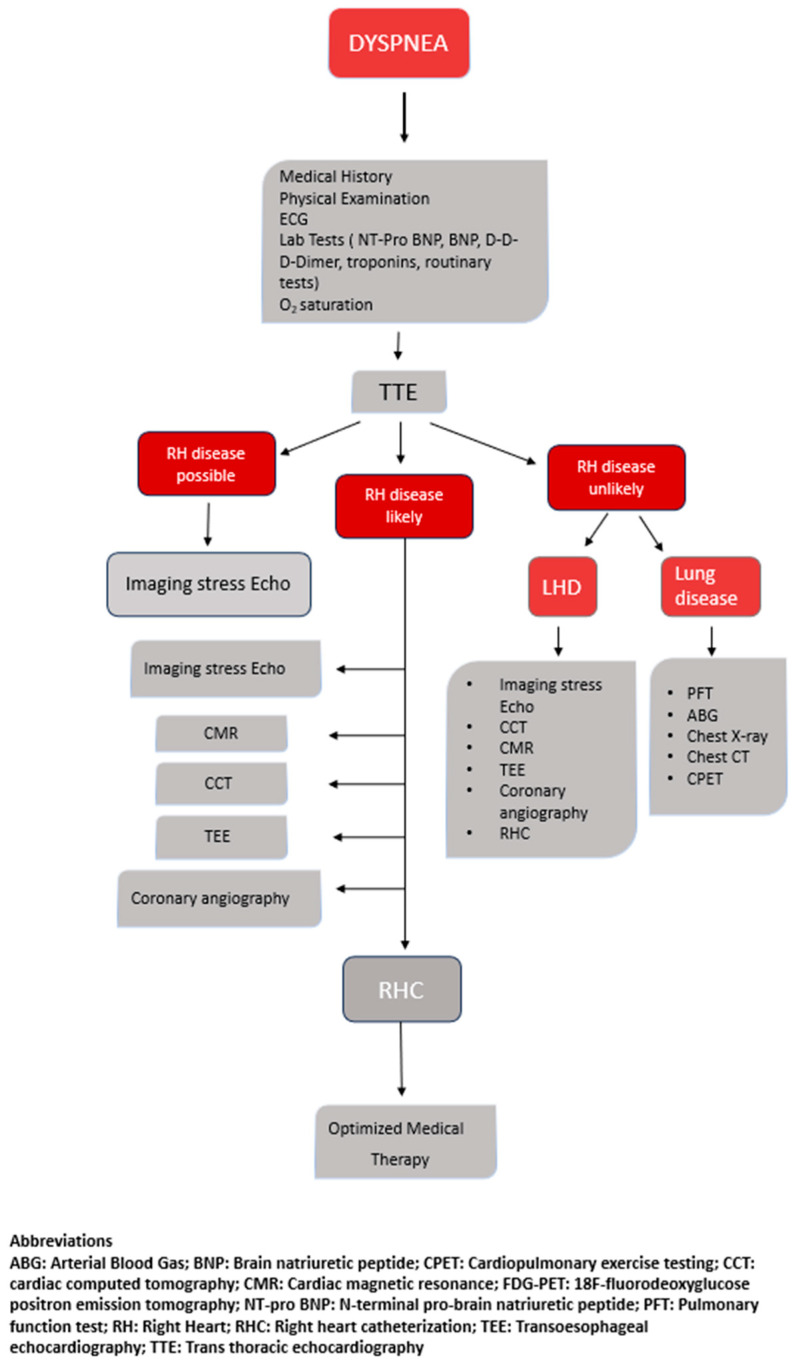
RH-PCU diagnostic algorithm.

As such, the stress imaging of the right heart is an emerging frontier with the potential to refine risk stratification and optimize management in a wide range of cardiopulmonary disorders (Figure 6 illustrates the integrated diagnostic approach to the RH-PCU). Furthermore, in the Supplementary Data, there is a description of which imaging modality to use in different clinical scenarios.

## Data Availability

Data presented in this study are available on request due to privacy and ethical restrictions.

## Supplementary Materials

The following supporting information can be downloaded at: https://www.mdpi.com/article/10.3390/life15081194/s1, Table S1: Description of which imaging modality to use in different clinical scenarios.

## Abbreviations

The following abbreviations are used in this manuscript:

ABG	Arterial Blood Gas
ARDS	Acute Respiratory Distress Syndrome
BNP	B-type Natriuretic Peptide
CHD	Coronary Heart Disease/Congenital Heart Disease (ambiguità)
CMR	Cardiac Magnetic Resonance
CMR-FT	Cardiac Magnetic Resonance Feature Tracking
CO	Cardiac Output
COPD	Chronic Obstructive Pulmonary Disease
CT	Computed Tomography
CTEPH	Chronic Thromboembolic Pulmonary Hypertension
DCM	Dilated Cardiomyopathy
DVT	Deep Vein Thrombosis
ECG	Electrocardiogram
ECV	Extracellular Volume
EF	Ejection Fraction
FAC	Fractional Area Change
HCM	Hypertrophic Cardiomyopathy
HF	Heart Failure
LA	Left Atrium
LAD	Left Anterior Descending (artery)
LGE	Late Gadolinium Enhancement
LUS	Lung Ultrasound
LV	Left Ventricle
LVEF	Left Ventricular Ejection Fraction
MRI	Magnetic Resonance Imaging
NT-proBNP	N-terminal pro-B-type Natriuretic Peptide
PA	Pulmonary Artery
PAH	Pulmonary Arterial Hypertension
PAP	Pulmonary Arterial Pressure
PASP	Pulmonary Artery Systolic Pressure
PCWP	Pulmonary Capillary Wedge Pressure
PH	Pulmonary Hypertension
PNX	Pneumothorax
PVR	Pulmonary Vascular Resistance
RA	Right Atrium
RAP	Right Atrial Pressure
RCM	Restrictive Cardiomyopathy
RH-PCU	Right Heart–Pulmonary Circulation Unit
RHC	Right Heart Catheterization
RIMP	Right Ventricular Index of Myocardial Performance
RV	Right Ventricle
RVEF	Right Ventricular Ejection Fraction
RVH	Right Ventricular Hypertrophy
RVMI	Right Ventricular Myocardial Infarction
RVMW	Right Ventricular Myocardial Work
SCD	Sudden Cardiac Death
SaO2	Arterial Oxygen Saturation
SvO2	Mixed Venous Oxygen Saturation
S′	RV Systolic Wave Velocity
TAPSE	Tricuspid Annular Plane Systolic Excursion
TDE	Transthoracic Doppler Echocardiography
TRV	Tricuspid Regurgitation Velocity
VHD	Valvular Heart Disease
mPAP	Mean Pulmonary Artery Pressure
sPAP	Systolic Pulmonary Artery Pressure

## References

[1] Haddad F., Doyle R., Murphy D.J., Hunt S.A. (2008). Right ventricular function in cardiovascular disease, part II: Pathophysiology, clinical importance, and management of right ventricular failure. Circulation.

[2] Ferrara F., Gargani L., Ostenfeld E., D’Alto M., Kasprzak J., Voilliot D., Selton-Suty C., Vriz O., Marra A.M., Argiento P. (2017). Imaging the right heart pulmonary circulation unit: Insights from advanced ultrasound techniques. Echocardiography.

[3] Lichtenstein D. (2014). Lung ultrasound in the critically ill. Ann. Intensive Care.

[4] Volpicelli G., Elbarbary M., Blaivas M., Lichtenstein D.A., Mathis G., Kirkpatrick A.W., Melniker L., Gargani L., Noble V.E., Via G. (2012). International evidence-based recommendations for point-of-care lung ultrasound. Intensive Care Med..

[5] Volpicelli G., Caramello V., Cardinale L., Mussa A., Bar F., Frascisco M.F. (2008). Bedside ultrasound of the lung for the monitoring of acute decompensated heart failure. Am. J. Emerg. Med..

[6] Gargani L., Volpicelli G. (2014). How I do it: Lung ultrasound. Cardiovasc. Ultrasound.

[7] Picano E., Scali M.C., Ciampi Q., Lichtenstein D. (2018). Lung Ultrasound for the Cardiologist. JACC Cardiovasc. Imaging.

[8] Bossone E., Dellegrottaglie S., Patel S., Grunig E., D’Andrea A., Ferrara F., Gargiulo P., D’Alto M., Soricelli A., Cittadini A. (2015). Multimodality imaging in pulmonary hypertension. Can. J. Cardiol..

[9] Contractor S., Maldjian P.D., Sharma V.K., Gor D.M. (2002). Role of helical CT in detecting right ventricular dysfunction secondary to acute pulmonary embolism. J. Comput. Assist. Tomogr..

[10] Wittram C., Kalra M.K., Maher M.M., Greenfield A., McLoud T.C., Shepard J.A. (2006). Acute and chronic pulmonary emboli: Angiography-CT correlation. AJR Am. J. Roentgenol..

[11] Nadrous H.F., Pellikka P.A., Krowka M.J., Swanson K.L., Chaowalit N., Decker P.A., Ryu J.H. (2005). Pulmonary hypertension in patients with idiopathic pulmonary fibrosis. Chest.

[12] Vriz O., Motoji Y., Ferrara F., Bossone E., Naeije R. (2018). The Right Heart-Pulmonary Circulation Unit in Systemic Hypertension. Heart Fail. Clin..

[13] Harjola V.P., Mebazaa A., Čelutkienė J., Bettex D., Bueno H., Chioncel O., Crespo-Leiro M.G., Falk V., Filippatos G., Gibbs S. (2016). Contemporary management of acute right ventricular failure: A statement from the Heart Failure Association and the Working Group on Pulmonary Circulation and Right Ventricular Function of the European Society of Cardiology. Eur. J. Heart Fail..

[14] Filippetti L., Voilliot D., Bellino M., Citro R., Go Y.Y., Lancellotti P. (2018). The Right Heart-Pulmonary Circulation Unit and Left Heart Valve Disease. Heart Fail. Clin..

[15] D’Andrea A., Formisano T., La Gerche A., Cardim N., Carbone A., Scarafile R., Martone F., D’Alto M., Bossone E., Galderisi M. (2018). Right Heart-Pulmonary Circulation Unit in Cardiomyopathies and Storage Diseases. Heart Fail. Clin..

[16] Pucci M., Iadevaia V., Gammaldi V., Iervolino A., Capece L.M., Sciascia D., Cuomo V., Iacono M., Paoletta D., Santoro C. (2023). Right Ventricular Myocardial Involvement in Anderson-Fabry Disease at Diagnosis: Evaluation with Three-Dimensional Strain Imaging. Life.

[17] Femia G., French J.K., Juergens C., Leung D., Lo S. (2021). Right ventricular myocardial infarction: Pathophysiology, clinical implications and management. Rev. Cardiovasc. Med..

[18] Surkova E., Cosyns B., Gerber B., Gimelli A., la Gerche A., Ajmone M.N. (2022). The dysfunctional right ventricle: The importance of multi-modality imaging. Eur. Heart J. Cardiovasc. Imaging.

[19] Ho S.Y., Nihoyannopoulos P. (2006). Anatomy, echocardiography, and normal right ventricular dimensions. Heart.

[20] Le Tourneau T., Piriou N., Donal E., Deswarte G., Topilsky Y., Lamblin N., Warin-Fresse K., Crochet D., Damy T., Trochu J.N. (2011). Imaging and modern assessment of the right ventricle. Minerva Cardioangiol..

[21] Lang R.M., Badano L.P., Mor-Avi V., Afilalo J., Armstrong A., Ernande L., Flachskampf F.A., Foster E., Goldstein S.A., Kuznetsova T. (2015). Recommendations for cardiac chamber quantifcation by echocardiography in adults: An update from the American Society of Echocardiography and the European Association of Cardiovascular Imaging. J. Am. Soc. Echocardiogr..

[22] Damy T., Kallvikbacka-Bennett A., Goode K., Khaleva O., Lewinter C., Hobkirk J., Nikitin N.P., Dubois-Randé J.-L., Hittinger L., Clark A.L. (2012). Prevalence of, Associations with, and Prognostic Value of Tricuspid Annular Plane Systolic Excursion (TAPSE) Among Out-Patients Referred for the Evaluation of Heart Failure. J. Card. Fail..

[23] Aloia E., Cameli M., D’Ascenzi F., Sciaccaluga C., Mondillo S. (2016). TAPSE: An old but useful tool in different diseases. Int. J. Cardiol..

[24] Lang R.M., Badano L.P., Tsang W., Adams D.H., Agricola E., Buck T., Faletra F.F., Franke A., Hung J., De Isla L.P. (2012). EAE/ASE recommendations for image acquisition and display using three-dimensional echocardiography. Eur. Heart J. Cardiovasc. Imaging.

[25] Otani K., Nabeshima Y., Kitano T., Takeuchi M. (2020). Accuracy of fully automated right ventricular quantification software with 3D echocardiography: Direct comparison with cardiac magnetic resonance and semi-automated quantification software. Eur. Heart J. Cardiovasc. Imaging.

[26] Voigt J.-U., Cvijic M. (2019). 2- and 3-Dimensional Myocardial Strain in Cardiac Health and Disease. JACC Cardiovasc. Imaging.

[27] Kalam K., Otahal P., Marwick T.H. (2014). Prognostic implications of global LV dysfunction: A systematic review and meta-analysis of global longitudinal strain and ejection fraction. Heart.

[28] Mondillo S., Galderisi M., Mele D., Cameli M., Lomoriello V.S., Zacà V., Ballo P., D’ANdrea A., Muraru D., Losi M. (2011). Speckle-tracking echocardiography: A new technique for assessing myocardial function. J. Ultrasound Med..

[29] Butcher S.C., Fortuni F., Montero-Cabezas J.M., Abou R., El Mahdiui M., van der Bijl P., van der Velde E.T., Ajmone Marsan N., Bax J.J., Delgado V. (2021). Right ventricular myocardial work: Proof-of-concept for non-invasive assessment of right ventricular function. Eur. Heart J. Cardiovasc. Imaging.

[30] Wu J., Huang X., Chen W., Tang Y., Chen X., Wang X., Jing B., Sun Y., Huang K., Gao Q. (2023). Noninvasive right ventricular work in patients with atrial septal defects: A proof-of-concept study. Cardiovasc. Ultrasound.

[31] DiLorenzo M.P., Bhatt S.M., Mercer-Rosa L. (2015). How best to assess right ventricular function by echocardiography. Cardiol. Young.

[32] Panesar D.K., Burch M. (2017). Assessment of Diastolic Function in Congenital Heart Disease. Front. Cardiovasc. Med..

[33] Rudski L.G., Gargani L., Armstrong W.F., Lancellotti P., Lester S.J., Grünig E., D’Alto M., Åström Aneq M., Ferrara F., Saggar R. (2018). Stressing the Cardiopulmonary Vascular System: The Role of Echocardiography. J. Am. Soc. Echocardiogr..

[34] Rudski L.G., Lai W.W., Afilalo J., Hua L., Handschumacher M.D., Chandrasekaran K., Solomon S.D., Louie E.K., Schiller N.B. (2010). Guidelines for the echocardiographic assessment of the right heart inadults: A report from the American Society of Echocardiography endorsed by the European Association of Echocardiography, a registered branch of the European Society of Cardiology, and the Canadian Society of Echocardiography. J. Am. Soc. Echocardiogr..

[35] Ho J.E., Zern E.K., Lau E.S., Wooster L., Bailey C.S., Cunningham T., Eisman A.S., Hardin K.M., Farrell R., Sbarbaro J.A. (2020). Exercise Pulmonary Hypertension Predicts Clinical Outcomes in Patients with Dyspnea on Effort. J. Am. Coll. Cardiol..

[36] Bidart C.M., Abbas A.E., Parish J.M., Chaliki H.P., Moreno C.A., Lester S.J. (2007). The noninvasive evaluation of exercise-induced changes in pulmonary artery pressure and pulmonary vascular resistance. J. Am. Soc. Echocardiogr..

[37] Bossone E., Rubenfire M., Bach D.S., Ricciardi M., Armstrong W.F. (1999). Range of tricuspid regurgitation velocity at rest and during exercise in normal adult men: Implications for the diagnosis of pulmonary hypertension. J. Am. Coll. Cardiol..

[38] Argiento P., Chesler N., Mulè M., D’Alto M., Bossone E., Unger P., Naeije R. (2010). Exercise stress echocardiography for the study of the pulmonary circulation. Eur. Respir. J..

[39] Varga A., Garcia M.A., Picano E. (2006). International Stress Echo Complication Registry. Safety of stress echocardiography (from the International Stress Echo Complication Registry). Am. J. Cardiol..

[40] Gargani L., Pugliese N.R., De Biase N., Mazzola M., Agoston G., Arcopinto M., Argiento P., Armstrong W.F., Bandera F., Cademartiri F. (2023). RIGHT Heart International NETwork (RIGHT-NET) Investigators. Exercise Stress Echocardiography of the Right Ventricle and Pulmonary Circulation. J. Am. Coll. Cardiol..

[41] Badano L.P., Addetia K., Pontone G., Torlasco C., Lang R.M., Parati G., Muraru D. (2020). Advanced imaging of right ventricular anatomy and function. Heart.

[42] Syed M.A., Raman S.V., Simonetti O.P. (2015). Basic Principles of Cardio Vascular MRI: Physics and Imaging Techniques.

[43] Swift A.J., Rajaram S., Condliffe R., Capener D., Hurdman J., AElliot C., Wild J.M., Kiely D.G. (2012). Diagnostic accuracy of cardiovascular mag netic resonance imaging of right ventricular morphology and function in the assessment of suspected pulmonary hypertension results from the ASPIRE registry. J. Cardiovasc. Magn. Reson..

[44] Dellegrottaglie S., Sanz J., Poon M., Viles-Gonzalez J.F., Sulica R., Goyenechea M., Macaluso F., Fuster V., Rajagopalan S. (2007). Pulmonary hypertension: Accuracy of detection with left ventricular septal-to-free wall curvature ratio measured at cardiac MR. Radiology.

[45] Boerrigter B., Mauritz G.J., Marcus J.T., Helderman F., Postmus P.E., Westerhof N., Vonk-Noordegraaf A. (2010). Progressive dilatation of the main pul monary artery in pulmonary arterial hypertension is irrespec tive of changes in pulmonary artery pressure. B59. Pulmonary Arterial Hypertension: Diagnosis, Hemodynamic Assessment, and Imaging.

[46] Doyle C.M., Orr J., Greenwood J.P., Plein S., Tsoumpas C., Bissell M.M. (2021). Four-dimensional flow magnetic resonance imaging in the assessment of blood flow in the heart and great vessels: A systematic review. J. Magn. Reason. Imaging.

[47] Markl M., Kilner P.J., Ebbers T. (2011). Comprehensive 4D velocity mapping of the heart and great vessels by cardiovascular magnetic resonance. J. Cardiovasc. Magn. Reason..

[48] Azarine A., Garcon P., Stansal A., Canepa N., Angelopoulos G., Silvera S., Sidi D., Marteau V., Zins M. (2019). Four-dimensional Flow MRI: Principles and Cardiovascular Applications. Radiographics.

[49] Zhao X., Leng S., Tan R.-S., Chai P., Yeo T.J., Bryant J.A., Teo L.L.S., Fortier M.V., Ruan W., Low T.T. (2022). Right ventricular energetic biomarkers from 4D Flow CMR are associated with exertional capacity in pulmonary arterial hypertension. J. Cardiovasc. Magn. Reson..

[50] Scatteia A., Baritussio A., Bucciarelli-Ducci C. (2017). Strain imaging using cardiac magnetic resonance. Heart Fail. Rev..

[51] Pedrizzetti G., Claus P., Kilner P.J., Nagel E. (2016). Principles of cardiovascular magnetic resonance feature tracking and echocardiographic speckle tracking for informed clinical use. J. Cardiovasc. Magn. Reson..

[52] Morais P., Marchi A., Bogaert J.A., Dresselaers T., Heyde B., D’hooge J., Bogaert J. (2017). Cardiovascular magnetic resonance myocardial feature tracking using a non-rigid, elastic image registration algorithm: Assessment of variability in a real-life clinical setting. J. Cardiovasc. Magn. Reson..

[53] de Siqueira M.E.M., Pozo E., Fernandes V.R., Sengupta P.P., Modesto K., Gupta S.S., Barbeito-Caamaño C., Narula J., Fuster V., Caixeta A. (2016). Characterization and clinical significance of right ventricular mechanics in pulmonary hypertension evaluated with cardiovascular magnetic resonance feature tracking. J. Cardiovasc. Magn. Reson..

[54] Aquaro G.D., Perfetti M., Camastra G., Monti L., Dellegrottaglie S., Moro C., Pepe A., Todiere G., Lanzillo C., Scatteia A. (2017). Cardiac MR with late gadolinium enhancement in acute myocarditis with preserved systolic function: ITAMY study. J. Am. Coll. Cardiol..

[55] Freed B.H., Gomberg-Maitland M., Chandra S., Mor-Avi V., Rich S., Archer S.L., Jamison E.B., Lang R.M., Patel A.R. (2012). Late gadolinium enhancement cardiovascular magnetic resonance predicts clinical worsening in patients with pulmonary hypertension. J. Cardiovasc. Magn. Reson..

[56] Swift A.J., Rajaram S., Capener D., Elliot C., Condliffe R., Wild J.M., Kiely D.G. (2014). LGE patterns in pulmonary hypertension do not impact overall mortality. JACC Cardiovasc. Imaging.

[57] Saunders L.C., Johns C.S., Stewart N.J., Oram C.J.E., Capener D.A., Puntmann V.O., Elliot C.A., Condliffe R.C., Kiely D.G., Graves M.J. (2018). Diagnostic and prognostic significance of cardiovascular magnetic resonance native myocardial T1 mapping in patients with pulmonary hypertension. J. Cardiovasc. Magn. Reson..

[58] Patel R.B., Li E., Benefield B.C., Swat S.A., Polsinelli V.B., Carr J.C., Shah S.J., Markl M., Collins J.D., Freed B.H. (2020). Diffuse right ventricular fibrosis in heart failure with preserved ejection fraction and pulmonary hypertension. ESC Heart Fail..

[59] Guo Y.K., Gao H.L., Zhang X.C., Wang Q.L., Yang Z.G., Ma E.S. (2010). Accuracy and reproducibility of assessing right ventricular function with 64 section multi-detector row CT: Comparison with magnetic resonance imaging. Int. J. Cardiol..

[60] Pickett C.A., Cheezum M.K., Kassop D., Villines T.C., Hulten E.A. (2015). Accuracy of cardiac CT, radionucleotide and invasive ventriculography, two- and three-dimensional echocardiography, and SPECT for left and right ventricular ejection fraction compared with cardiac MRI: A meta analysis. Eur. Heart. J. Cardiovasc. Imaging.

[61] Hahn R.T., Lerakis S., Delgado V., Addetia K., Burkhoff D., Muraru D., Pinney S., Friedberg M.K. (2023). Multimodality Imaging of Right Heart Function: JACC Scientific Statement. J. Am. Coll. Cardiol..

[62] Mehra M.R., Canter C.E., Hannan M.M., Semigran M.J., Uber P.A., Baran D.A., Danziger-Isakov L., Kirklin J.K., Kirk R., Kushwaha S.S. (2016). The 2016 International Society for Heart Lung Transplantation listing criteria for heart transplantation:a10-yearupdate. J. Heart Lung Transplant..

[63] Gonzalez-Hermosillo L.M., Cueto-Robledo G., Roldan-Valadez E., Graniel-Palafox L.E., Garcia-Cesar M., Torres-Rojas M.B., Romero-Martinez B., Castro-Escalante K.Y. (2022). Right Heart Catheterization (RHC): A Comprehensive Review of Provocation Tests and Hepatic Hemodynamics in Patients with Pulmonary Hypertension (PH). Curr. Probl. Cardiol..

