# Supplementary material for: Right Heart Evaluation: A Tough Challenge for Clinicians
Source: Life (Basel). 2025 Jul 27;15(8):1194. doi: 10.3390/life15081194 (PMC12387904; doi:10.3390/life15081194)
Supplement: Supplementary file 1 [file life-15-01194-s001.zip › life-3724324-supplementary.pdf]

Clinical Scenario	Main imaging Modalities	Key Findings
Pulmonary Embolism	CTPA, TTE	Thrombus, RV dysfunction
Pulmonary Arterial Hypertension	TTE, CMR, V/Q SCAN, RHC	High sPAP, RV dilatation, perfusion defect
CTEPH	V/Q scan, CTPA, TTE, RHC	Perfusion mismatch, organized Thrombi
Right HF from LHD	TTE, Chest X-Ray	RV dysfunction, congestion

Abbreviations
CMR= cardiac magnetic resonance ; CTPA= computed tomography pulmonary angiogram; CTEPH= chronic thrombo-embolic pulmonary hypertension; HF= Heart failure; RHC= Right Heart Catheterization; sPAP= = systolic pulmonary arterial pressure; RV= right ventricular; TTE= transthoracic echocardiography.

Table S1: Description of which imaging modality to use in different clinical scenarios
